# Supplementary material for: Dysregulation of REV-ERBα impairs GABAergic function and promotes epileptic seizures in preclinical models
Source: Nat Commun. 2021 Feb 22;12:1216. doi: 10.1038/s41467-021-21477-w (PMC7900242; doi:10.1038/s41467-021-21477-w)
Supplement: Supplementary file 1 — Supplementary information [file 41467_2021_21477_MOESM1_ESM.pdf]

Supplementary Data

Manuscript title:

Dysregulation of REV-ERBα impairs GABAergic function and promotes epileptic seizures in preclinical models

Supplementary Table 1. Intrinsic properties of dentate gyrus granule cells.

Group	Input resistance（MΩ）	Resting membrane potential（mV）	Rheobase current（pA）	Firing frequency（Hz at 100 pA）
WT	350.6 ± 19.8	-77.4 ± 1.9	51.8 ± 6.7	17.2 ± 3.1
KO	340.5 ± 20.1	-75.7 ± 1.6	54.8 ± 6.0	19.9 ± 2.5
Vehicle	345.6 ± 33.8	-74.8 ± 2.7	53.6 ± 10.9	18.8 ± 3.7
SR8278	347.5 ± 28.5	-77.3 ± 2.4	58.3 ± 6.8	18.4 ± 3.8

Supplementary Table 2. Information for clinical patients.

Subject	Gender	Diagnosis	Age of onset (range)	Age at surgery (range)	AEDs
P1	Female	TLE	2-26	20-67	Oxcarbazepine
P2	Female	TLE	2-26	20-67	Oxcarbazepine
P3	Male	TLE	2-26	20-67	Valproate, Oxcarbazepine
P4	Female	TLE	2-26	20-67	Oxcarbazepine
P5	Female	TLE	2-26	20-67	Oxcarbazepine
P6	Male	TLE	2-26	20-67	Oxcarbazepine
P7	Female	TLE	2-26	20-67	Valproate, Oxcarbazepine
P8	Female	TLE	2-26	20-67	Oxcarbazepine
P9	Female	TLE	2-26	20-67	Valproate, Oxcarbazepine
P10	Female	TLE	2-26	20-67	Oxcarbazepine
C1	Male	Glioma		20-67	
C2	Female	Glioma		20-67	
C3	Male	Glioma		20-67	
C4	Female	Glioma		20-67	
C5	Male	Glioma		20-67	

Supplementary Table 3. Oligonucleotides used in this study.

	Forward (5'-3' sequence)	Reverse (5'-3' sequence)
siRNA		
siRev-erbα	CUUCGUUGUUCAACGUGAATT	UUCACGUUGAACAACGAAGTT
siE4bp4	GCACAAGCUUCGGAUUAAATT	UUUAAUCCGAAGCUUGUGCTT
siNC	UUCUCCGAACGUGUCACGUTT	ACGUGACACGUUCGGAGAATT
ChIP		
mSlc6a1_E4bp4	AACAACTTACATCCCTCCT	GCAAATGTGACTAGAGCC
mSlc6a11_E4bp4	TGTCTAAGGGTCACAGGG	ACCGACAGATGGAGGAAG
Non-specific region	GGCTTGTCCTGTTGTATGA	GAGTGCTATCTCCGATGC

Supplementary Table 4. Primer sequences for quantitative real-time PCR (qPCR).

Gene	Forward (5'-3' sequence)	Reverse (5'-3' sequence)
mRev-erbα	TTTTTCGCCGGAGCATCCAA	ATCTCGGCAAGCATCCGTTG
mE4bp4	CTTTCAGGACTACCAGACATCCAA	GATGCAACTTCCGGCTACCA
mSlc6a1	GAAAGCTGTCTGATTCTGAGGTG	AGCAAACGATGATGGAGTCCC
mSlc6a11	TGTTGAGCGTAGCTGGAGAGA	AGCAGATGAAAAACACCACGTA
mGabra5	TGACCCAAACCCTCCTTGTCT	GTGATGTTGTCATTGGTCTCGT
mGabrd	ATTGGGGACTACGTGGGCT	CCACATTCACAGGAGCACC
mmGlur2	GCTCCCACAGCTATCACCG	TCATAACGGGACTTGTCGCTC
mmGlur3	CTGGAGGCCATGTTGTTTGC	CATCCACTTTAGTCAACGATGCT
mmGlur5	CCCAGCACAAGTCGGAAATAG	TGTCTGGTTGGGGTTCTCCTT
mGlur2	GGACTCGGGAAGTAAGGAAAAG	CACTCTCGATGCCATATACGTTG
mGlur3	ACCATCAGCATAGGTGGACTT	ACGTGGTAGTTCAAATGGAAGG
mNkcc1	TTCCGCGTGAACTTCGTGG	TTGGTGTGGGTGTCATAGTAGT
mKcc1	ATGCCTCACTTCACCGTGG	GTTACCCTGTCCGTCCGAG
mGluk1	TGACATTGAGCAGGCTTTTTGT	CGAATCCCCTTTTCCTCTCGTAA
mGluk2	AATCTAGTCTTCAGTCGCTCCA	TCCTTGCGAATATCCGATCCA
mGluk3	AGGTCCTAATGTCACTGACTCTC	TGCCATAAAGGGTCCTATCAGAC
mGluk4	CCCTGAGGATTGCTGCTATCT	CACCCTTGGGGAGGATCTGA
mGluk5	ATAGTCGCCTTCGCCAATCC	GTGTCCGTGGTCTCGTACTG
mGs	TGAACAAAGGCATCAAGCAAATG	CAGTCCAGGGTACGGGTCTT
mGlast	ACCAAAAGCAACGGAGAAGAG	GGCATTCCGAAACAGGTAACTC
mGlt-1	GCACGAGAGCTATGGTGTATTAC	GTTTGGGATTACCTGGGTGGA
mEaac1	CTTCCTACGGAATCACTGGCT	CGATCAGCGGCAAAATGACC
mEaat4	AGCAGCCACGGCAATAGTC	ATGCCAAGCTGACACCAATGA
mEaat5	CGCACAGTGTGTAAGCACAAC	GCATCCGCATCAAGAGTTCTC
mGabra4	ACAATGAGACTCACCATAAGTGC	GGCCTTTGGTCCAGGTGTAG
mGabra6	TGCCCAAGCTCAACTTGAAGA	GCCGTAGACGGTTGTCATAGC
mvGlut1	GGTGGAGGGGGTCACATAC	AGATCCCGAAGCTGCCATAGA
mClock	TCTGGATTCGCTGGCTAATGG	GACCTCCGCTGTGTCATCTT
mPer2	CCACACTTGCCTCCGAAATA	ACTGCCTCTGGACTGGAAGA
mDbp	ACATCTAGGGACACACCCAGTC	AAGTCTCATGGCCTGGAATG
mBmal1	CTCCAGGAGGCAAGAAGATTC	ATAGTCCAGTGGAAGGAATG
mCyclophilin b	TCCACACCCTTTTCCGGTCC	CAAAAGGAAGACGACGGAGC
hREV-ERBα	CCAACAACAACACAGGTGGCG	GGGGATGGTGGGAAGTAGGT
hBMAL1	TTAAGAGGTGCCACCAATCC	TTCCCTCGGTCACATCCTAC
hCLOCK	TGGGAATCCCTCAACTCAAC	GACTGAGGGAAGGTGCTCTG
hE4BP4	AGGGAAGCTGCAGAAGTCCTGAAA	AGTTGCTGGAGGATCGGTTGACTT
hDBP	GGCGTCGGGTGTTTTGGTTT	CTCCTTCTACAAGGTGGGCG
hGAPDH	CATGAGAAGTATGACAACAGCCT	AGTCCTTCCACGATACCAAAGT

m, mouse; h, human.

Supplementary Table 5. Antibodies used for Western blotting and immunohistochemistry.

Primary or Secondary	Antibody	Species	Dilution	Source	Cat No.	Application
1st	Rev-erbα	rabbit	1:1,000	Proteintech	14506-1-AP	WB
1st	Slc6a1	rabbit	1:500	Abclonal	A15099	WB
1st	Slc6a11	rabbit	1:1,000	Abclonal	A11702	WB
1st	Bmal1	rabbit	1:200	Abcam	ab3350	WB
1st	Dbp	rabbit	1:1,000	Abcam	ab22824	WB
1st	Clock	rabbit	1:200	Abcam	ab3517	WB
1st	E4bp4	rabbit	1:500	MBL	M225-3	WB
1st	Rev-erbα	rabbit	1:100	Abcam	ab174309	IHC
1st	GFAP	goat	1:500	Abcam	ab53554	IHC
1st	NeuN	mouse	1:500	Abcam	ab104224	IHC
1st	Iab1	goat	1:200	Abcam	ab48004	IHC
2nd	goat anti-rabbit IgG-HRP	rabbit	1:5,000	HuaBio	HA1001	WB
2nd	Alexa Fluor 488 Goat anti-Rabbit IgG (H+L)	rabbit	1:400	Thermo Fischer	A-11034	IHC
2nd	Alexa 555 Donkey anti-Mouse IgG (H+L)	mouse	1:200	Thermo Fischer	A-31570	IHC
2nd	Alexa 555 Donkey anti-Goat IgG (H+L)	goat	1:400	Thermo Fischer	A-21432	IHC

IHC, immunohistochemistry; H+L, heavy and light chain.

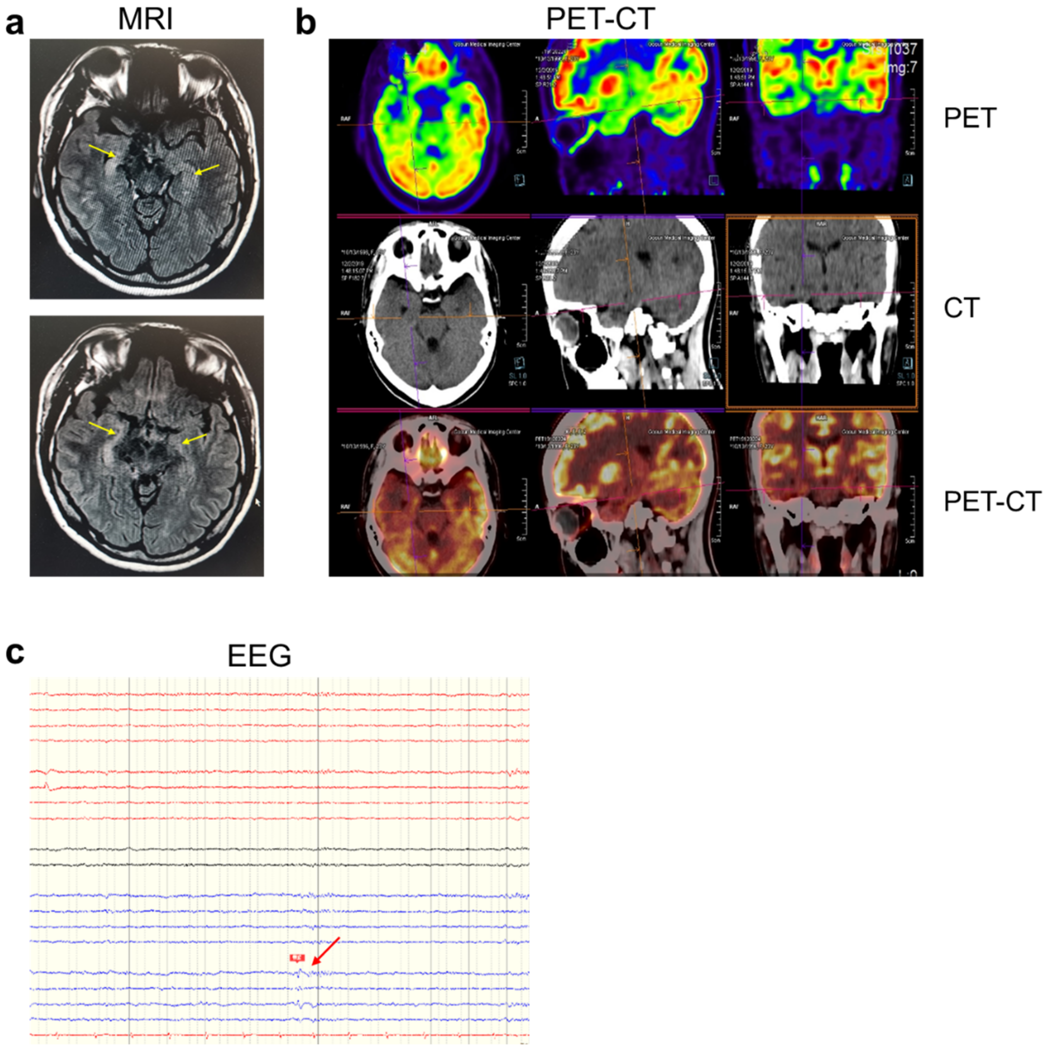

Supplementary Figure 1. **Epileptogenic foci of TLE patients were identified by brain MRI, PET-CT and EEG**. (**a**) Pre-surgical MRI, showing dysplasia in the mesial temporal lobe (yellow arrows). (**b**) PET-CT, showing hypometabolism in the mesial temporal lobe. (**c**) EEG tracings, showing spike-and-wave discharges (red arrow) at the temporal lobe. EEG, electroencephalography; MRI, magnetic Resonance Imaging; PET-CT, positron emission tomography-computed tomography; TLE, temporal lobe epilepsy.

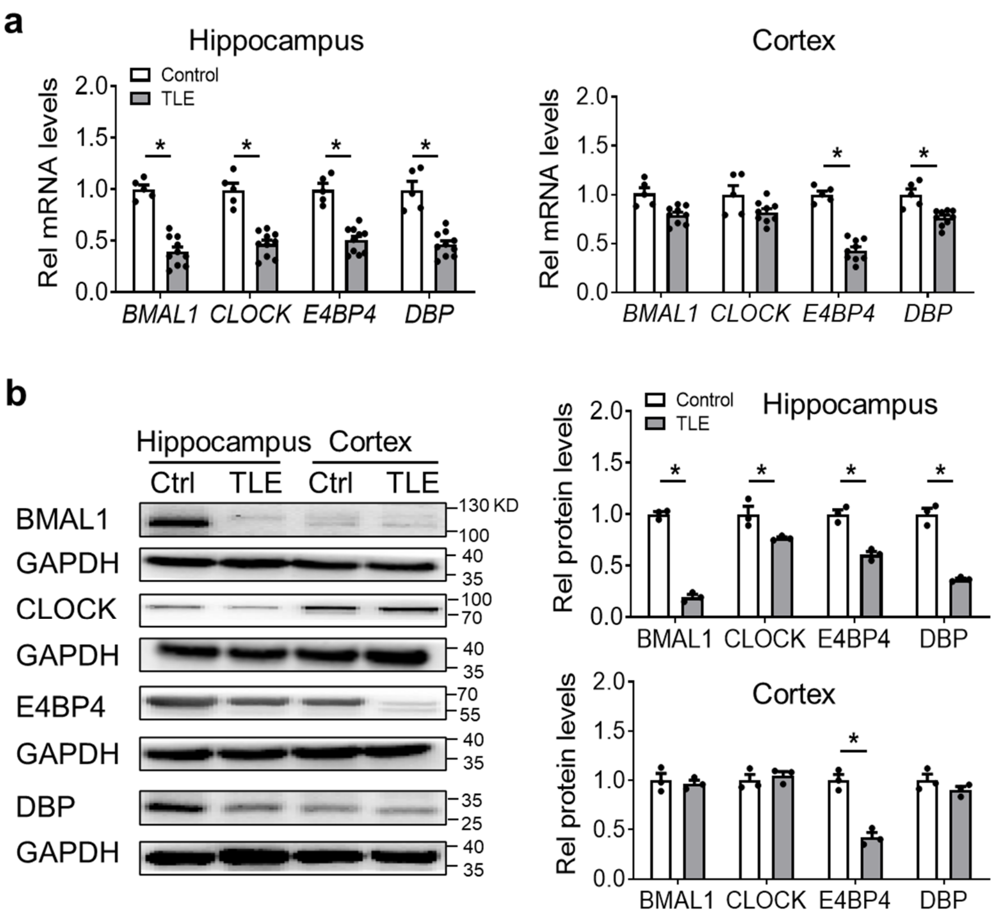

Supplementary Figure 2. **mRNA (a) and protein (b) expression of REV-ERBα target genes in hippocampus and temporal cortex from TLE patients (*n* = 10 biologically independent samples) and controls (*n* = 5 biologically independent samples)**. Western blot strips (a target protein and a loading control) were cut from one gel. *p* values (panel **a**, hippocampus, from left to right): < 0.0001, < 0.0001, < 0.0001, < 0.0001; *p* values (panel **a**, cortex, from left to right): 0.0025, 0.0496, < 0.0001, < 0.0001. *p* values (panel **b**, hippocampus, from left to right): 0.0118, 0.0436, 0.012, 0.0004; *p* values (panel **b**, cortex, from left to right): 0.7002, 0.5585, 0.0017, 0.2416. All data are presented as mean ± SEM. *p* values were obtained from two-sided t test. *represents a *p* value of < 0.05. Ctrl, control; Rel, relative; TLE, temporal lobe epilepsy.

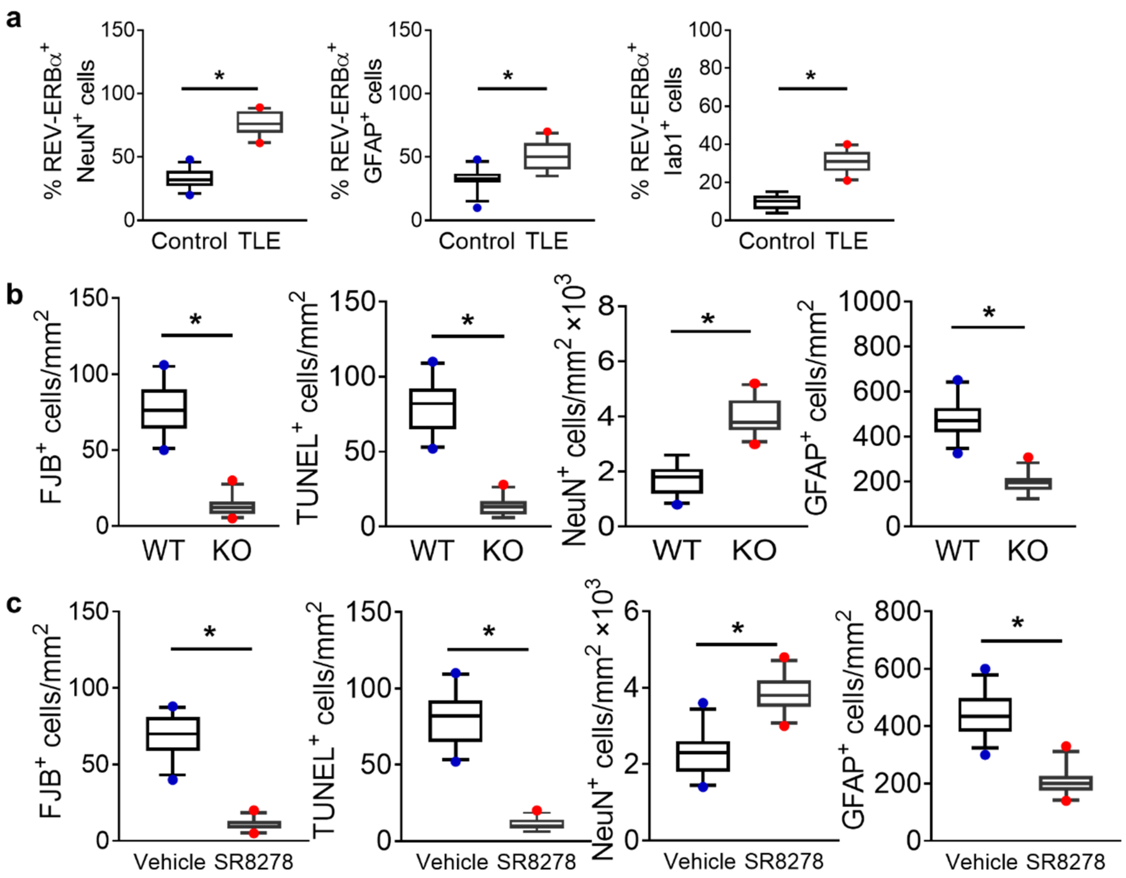

Supplementary Figure 3. **Quantitative data generated from immunofluorescence staining in this study.** (**a**) Quantification of REV-ERBα expression in $NeuN^+$, $GFAP^+$ and $Iba1^+$ cells of human epileptic and control tissues (related to Figure 1c-e in the main text) (*n* = 27 cells examined over three independent experiments). *p* values: < 0.0001, < 0.0001, < 0.0001, < 0.0001 (two-sided Mann-Whitney test). (**b**) FJB, TUNEL, NeuN and GFAP counts in the hippocampus of *Rev-erbα*$^{-/-}$ (KO) and wild-type (WT) mice 24 h after treatment with KA (related to Figure 2d-f in the main text) (*n* = 27 cells examined over three independent experiments). *p* values: < 0.0001, < 0.0001, < 0.0001, < 0.0001 (two-sided Mann-Whitney test). (**c**) FJB, TUNEL, NeuN and GFAP counts in the hippocampus of mice (pre-treated with SR8278 or vehicle) at 24 h after treatment with KA (related to Figure 3d-f in the main text) (*n* = 27 cells examined over three independent experiments). *p* values: < 0.0001, < 0.0001, < 0.0001, < 0.0001 (two-sided Mann-Whitney test). All data are shown as box-and-whisker with median (middle line), 25th-75th percentiles (box), and 5th and 95th percentile (whiskers) as well as outliers (single points). *represents a *p* value of < 0.05. FJB, Fluoro-Jade-B; TLE, temporal lobe epilepsy.

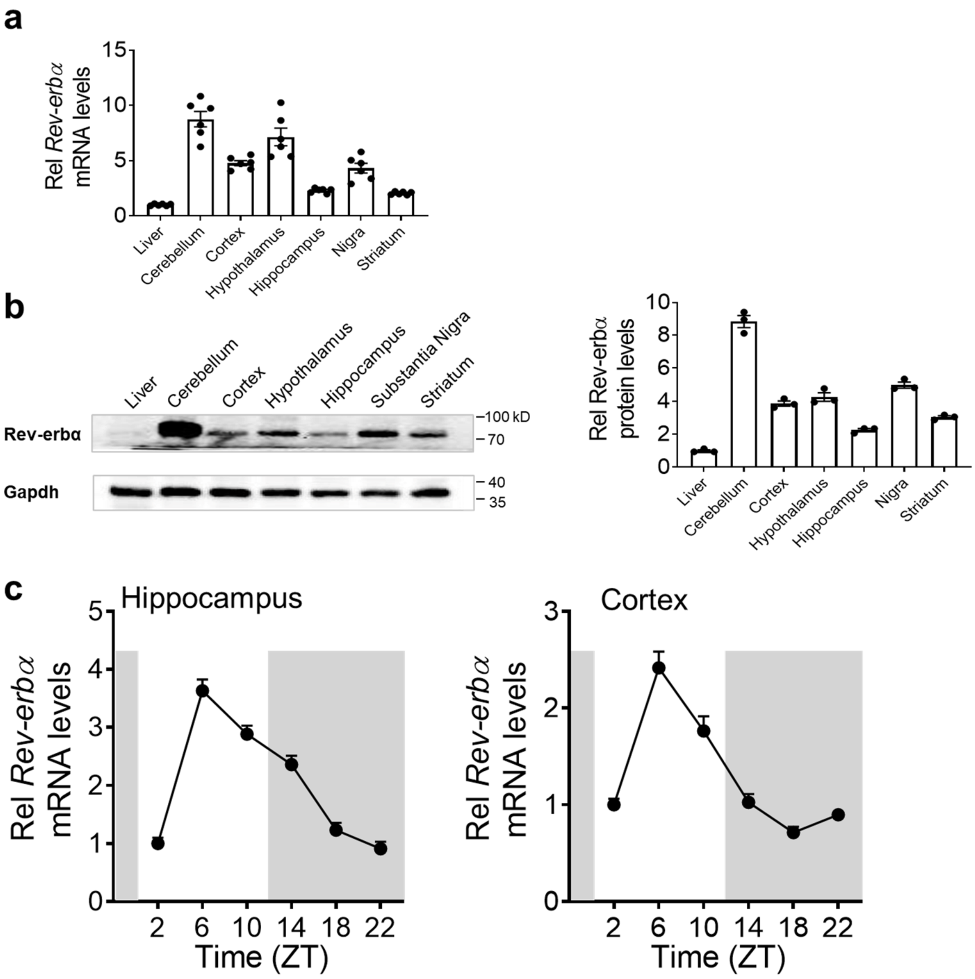

Supplementary Figure 4. **Rev-erbα expression in mouse brain.** (**a**) The mRNA expressions of *Rev-erbα* in the hippocampus, cortex, cerebellum, nigra, striatum, hypothalamus and liver of wild-type (WT) mice (*n* = 6 biologically independent samples). (**b**) Protein expressions of Rev-erbα in the hippocampus, cortex, cerebellum, nigra, striatum, hypothalamus and liver of WT mice. Each Western blot is representative of three independent experiments. Western blot strips (a target protein and a loading control) were cut from one gel. (**c**) Circadian (24-hour) rhythms of *Rev-erbα* mRNA in the hippocampus and cortex of WT mice (*n* = 6 mice per time point). All data are shown as mean ± SEM. Rel, relative; ZT, zeitgeber time.

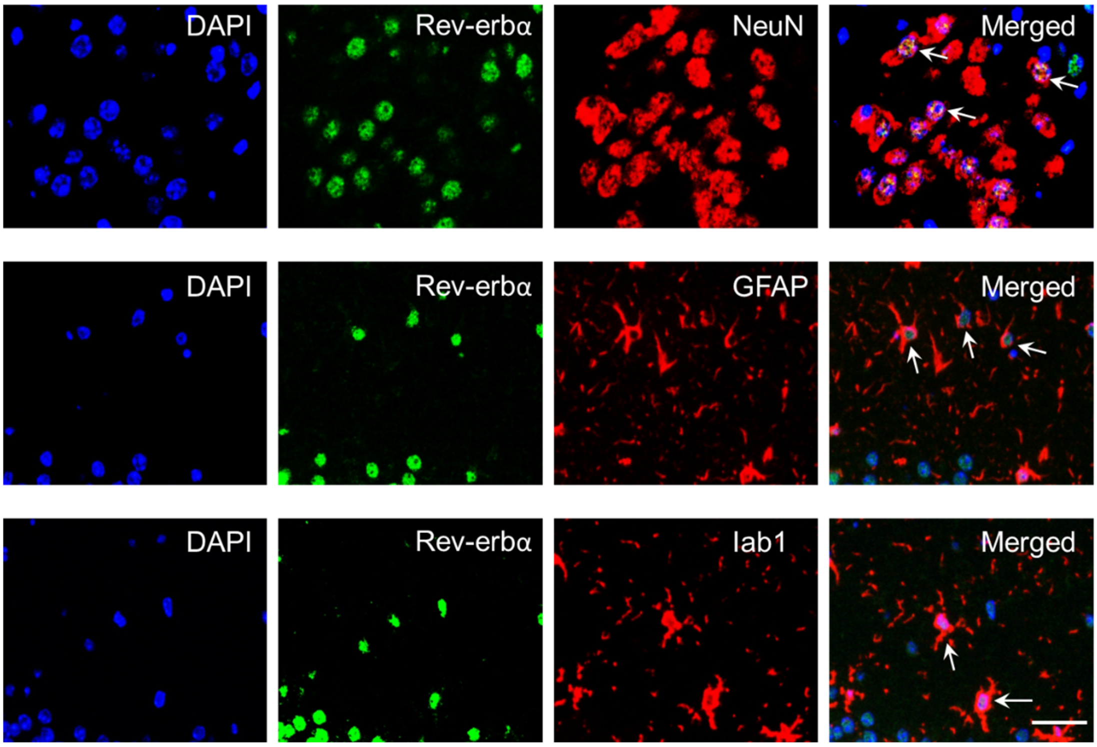

Supplementary Figure 5. **Double-labeled immunofluorescence staining showing co-localization of Rev-erbα with the neuronal marker NeuN, astrocytic marker GFAP and microglial marker Iba1 in the hippocampus of mice (white arrows).** Similar results were obtained in three independent experiments. Scale bar = 20 μm.

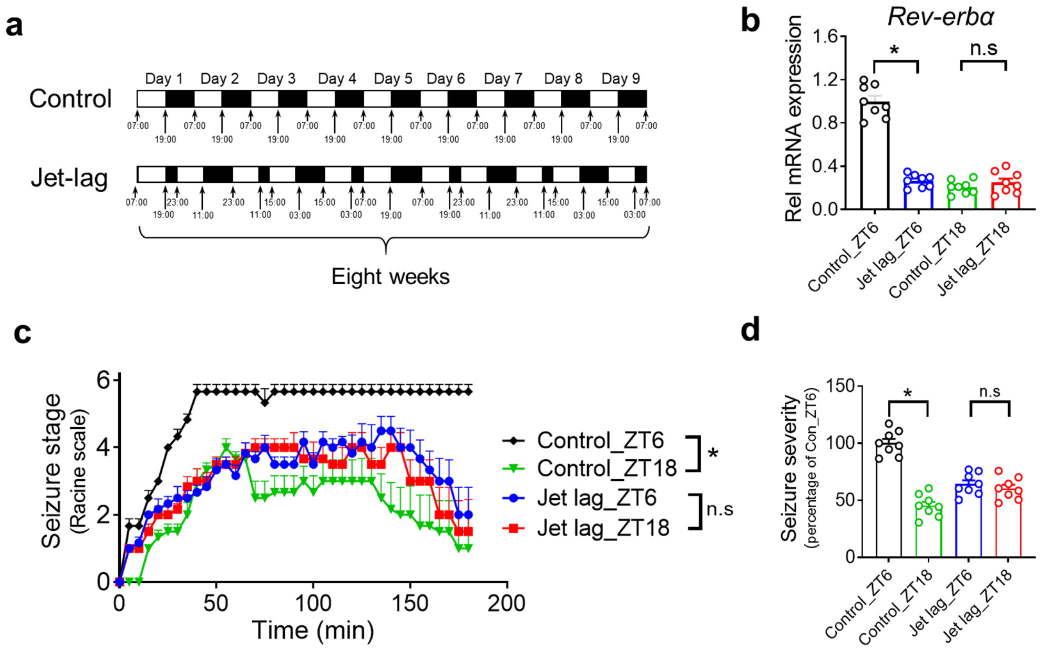

Supplementary Figure 6. **Effects of chronic jet lag on seizure severity in KA-induced acute seizure mice.** (**a**) Experimental scheme for establishment of chronic jet lag model. (**b**) mRNA expression of *Rev-erbα* in the hippocampus of jet-lagged and control mice at ZT6 and ZT18. Two-sided t test *p* values: < 0.0001 (ZT6) and 0.3185 (ZT18). (**c**) Seizure stages of jet-lagged and control mice injected with KA (20 mg/kg, i.p.) at ZT6 and ZT18. *p* values: 0.0035 (control) and 0.9926 (jet-lag) (Two-sided Kruskal-Wallis test). (**d**) Seizure severity of jet-lagged and control mice injected with KA (20 mg/kg, i.p.) at ZT6 and ZT18. Two-sided t test *p* values: 0.0031 (control) and 0.6958 (jet-lag). In panels b-d, data are presented as mean ± SEM, *n* = 8 mice per group. *represents a *p* value of < 0.05. n.s, no significant; ZT, zeitgeber time.

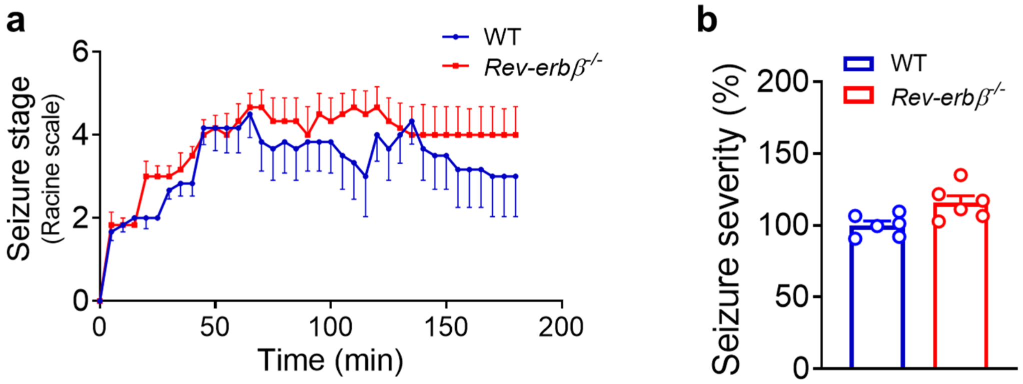

Supplementary Figure 7. Rev-erbβ ablation does not alter the seizure severity in kainic acid (KA)-induced acute seizure. (**a**) Seizure stages of *Rev-erbβ*$^{-/-}$ mice and wild-type littermates (WT) injected with KA (20 mg/kg, i.p.) at ZT6. (**b**) Seizure severity of *Rev-erbβ*$^{-/-}$ mice and wild-type littermates (WT) injected with KA (20 mg/kg, i.p.) at ZT6. All data are presented as mean ± SEM, *n* = 6 mice per group.

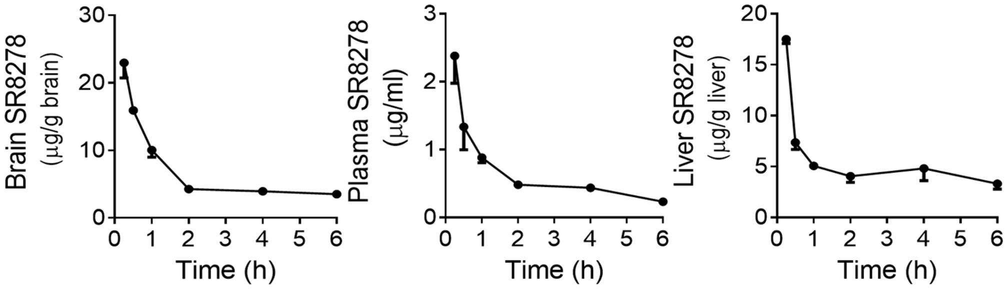

Supplementary Figure 8. **SR8278 concentration versus time profiles in the brain, liver and plasma after drug dosing to mice (25 mg/kg, i.p.).** Data were shown as mean ± SEM (*n* = 5 mice per time point).

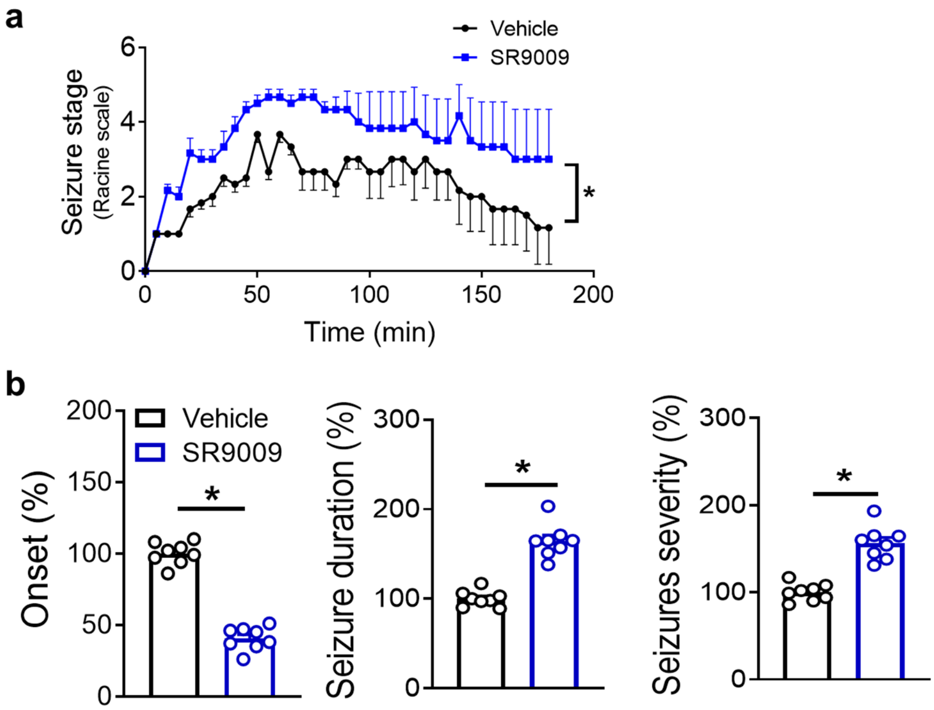

Supplementary Figure 9. Effects of SR9009 on seizure severity in KA-induced acute seizure mice. (**a**) Effects of SR9009 (Rev-erbα agonist, 50 mg/kg) on seizure stages of wild-type (WT) mice injected with KA (20 mg/kg, i.p.) at ZT18. p = 0.0288 (Two-sided Kruskal-Wallis test). (**b**) Effects of SR9009 on seizure parameters (onset, duration and severity) of WT mice injected with KA (20 mg/kg, i.p.) at ZT18. Two-sided t test p values: < 0.0001 (onset), < 0.0001 (seizure duration), and < 0.0001 (seizure severity). All data are mean ± SEM, n = 8 mice per group. *represents a p value of < 0.05.

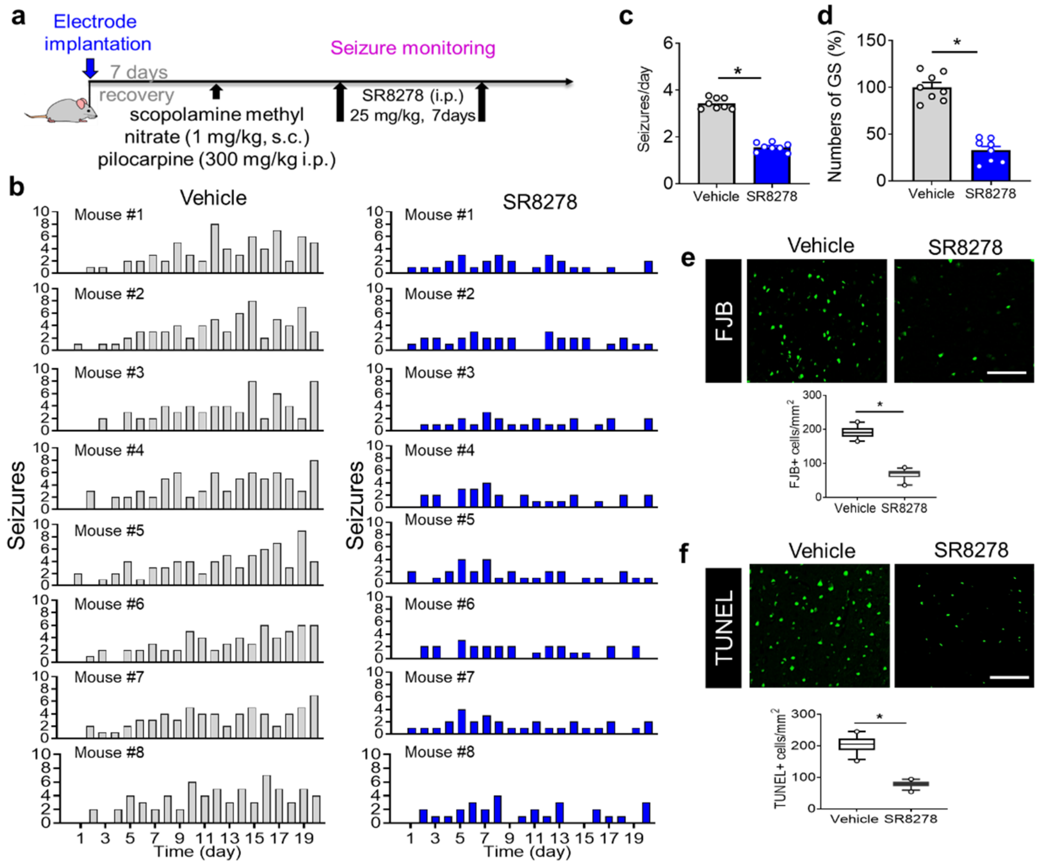

Supplementary Figure 10. Effects of SR8278 on chronic spontaneous seizures. (**a**) Experimental scheme for pilocarpine-induced chronic spontaneous seizures and SR8278 (25 mg/kg, i.p., injection at ZT6) treatment with wild-type (WT) mice. (**b**) The number of seizures for individual mouse in the following 20 days after pilocarpine treatment (n = 8 mice per group). (**c**) Effects of SR8278 on seizure frequency of WT mice injected with pilocarpine. Data are shown as mean ± SEM (n = 8 mice per group). $p < 0.0001$ (two-sided t test). (**d**) Effects of SR8278 on seizure severity [reflected by the numbers of generalized seizure (GS, stage ≥ 4)] in WT mice injected with pilocarpine. Data are shown as mean ± SEM (n = 8 mice per group). $p < 0.0001$ (two-sided t test). (**e**) FJB staining of hippocampus collected on the eighth day of SR8278 treatment (n = 27 biologically independent cells). $p < 0.0001$ (two-sided Mann-Whitney test). (**f**) TUNEL staining of hippocampus collected on the eighth day of SR8278 treatment (n = 27 biologically independent cells). $p < 0.0001$ (two-sided Mann-Whitney test). In lower panels of **e** and **f**, data are shown as box-and-whisker with median (middle line), 25th-75th percentiles (box), and 5th and 95th percentile (whiskers) as well as outliers (single points). *represents a p value of < 0.05. Scale bar = 50 μm.

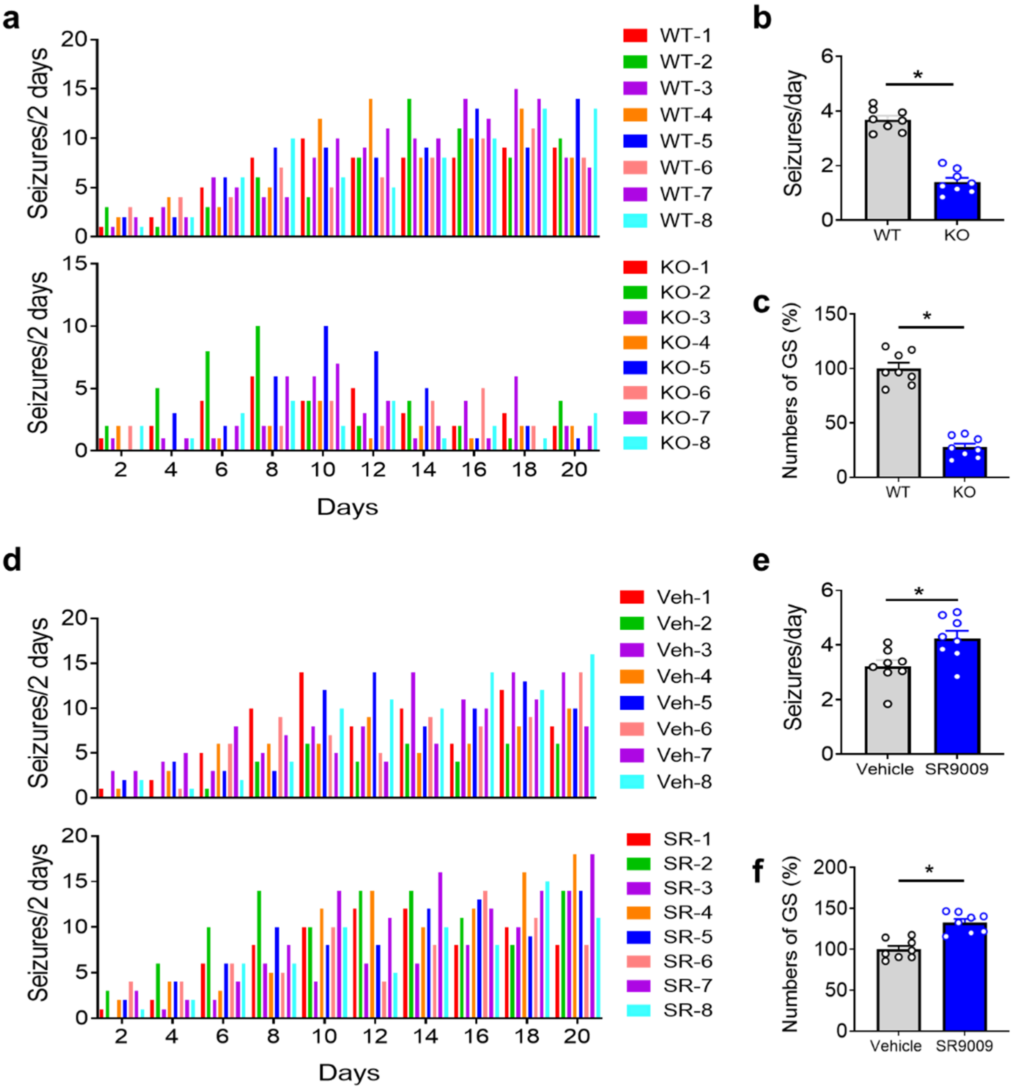

Supplementary Figure 11. Effects of Rev-erbα ablation or activation on chronic spontaneous seizures induced by pilocarpine. (**a**) The number of seizures for individual mouse in the following 20 days after pilocarpine treatment for *Rev-erbα*$^{-/-}$ (KO) mice and wild-type littermates (WT) (n = 8 mice per group). (**b**) Daily epileptic seizures of WT and *Rev-erbα*$^{-/-}$ mice after pilocarpine treatment (n = 8 mice per group). $p < 0.0001$ (two-sided t test). (**c**) Seizure severity [reflected by the numbers of generalized seizure (GS, stage ≥ 4)] in WT and *Rev-erbα*$^{-/-}$ (KO) mice after pilocarpine treatment. $p < 0.0001$ (two-sided t test). (**d**) Effect of SR9009 on the number of seizures for individual mouse in the following 20 days after pilocarpine treatment. (**e**) Effect of SR9009 on daily epileptic seizures of wild-type mice after pilocarpine treatment. $p < 0.0001$ (two-sided t test). (**f**) Effect of SR9009 on the seizure severity [reflected by the numbers of generalized seizure (GS, stage ≥ 4)] of wild-type mice after pilocarpine treatment. $p < 0.0001$ (two-sided t test). In panels b, c, e and f, data are shown as mean ± SEM, n = 8 mice per group. *represents a p value of < 0.05.

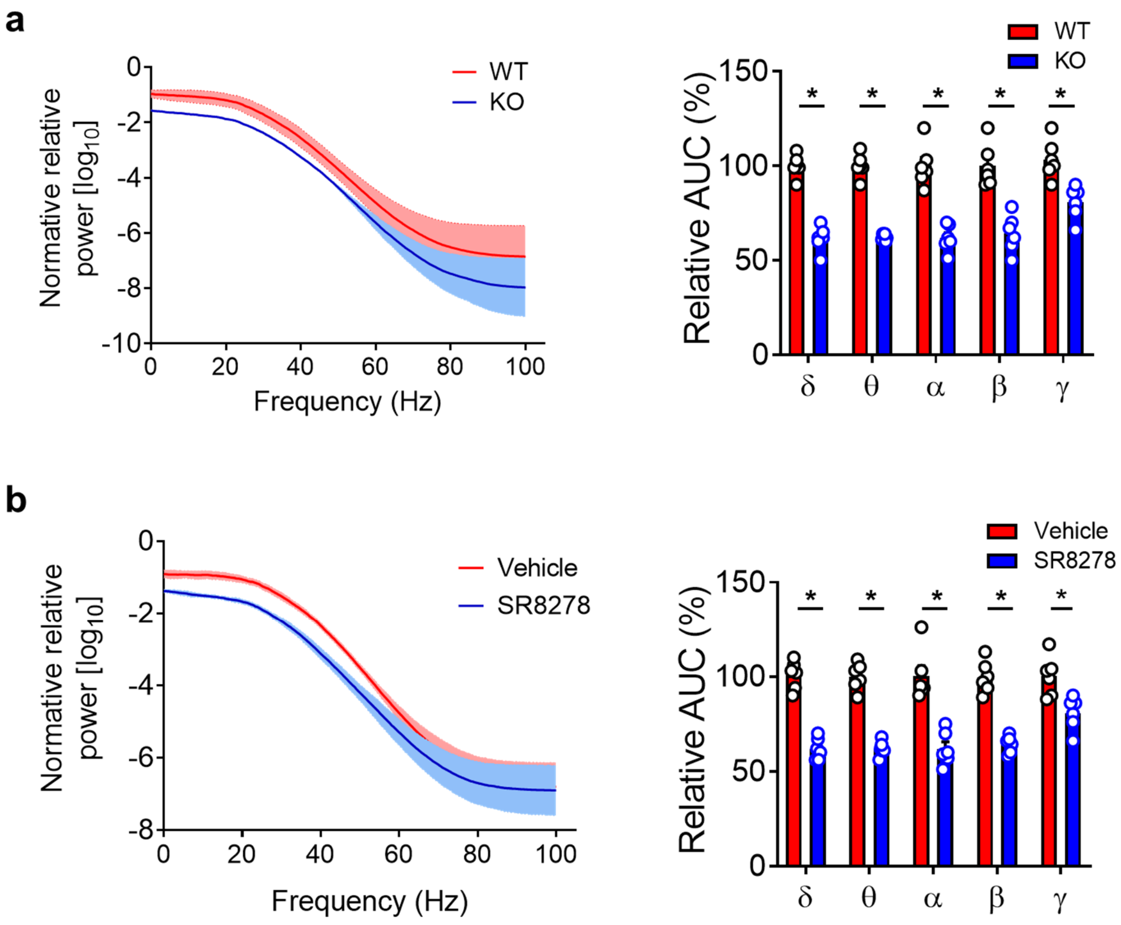

Supplementary Figure 12. Effects of Rev-erbα on frequency types in mice after kindling stimulations. (**a**) Frequency types in *Rev-erbα*$^{-/-}$ (KO) and wild-type (WT) mice after kindling stimulations (n = 6 mice per group). Two-sided t test p values: < 0.0001 (δ), < 0.0001 (θ), < 0.0001 (α), 0.0002 (β) and 0.0021 (γ). (**b**) Effects of SR8278 on frequency types in mice after kindling stimulations. Frequency bands: delta (0-3 Hz), theta (4-8 Hz), alpha (9-13 Hz), beta (14-30 Hz), and gamma (31-100 Hz). Two-sided t test p values: < 0.0001 (δ), < 0.0001 (θ), 0.0002 (α), < 0.0001 (β) and 0.006 (γ). Data are mean ± SEM, n = 6 mice per group. *represents a p value of < 0.05. AUC, area under the curve.

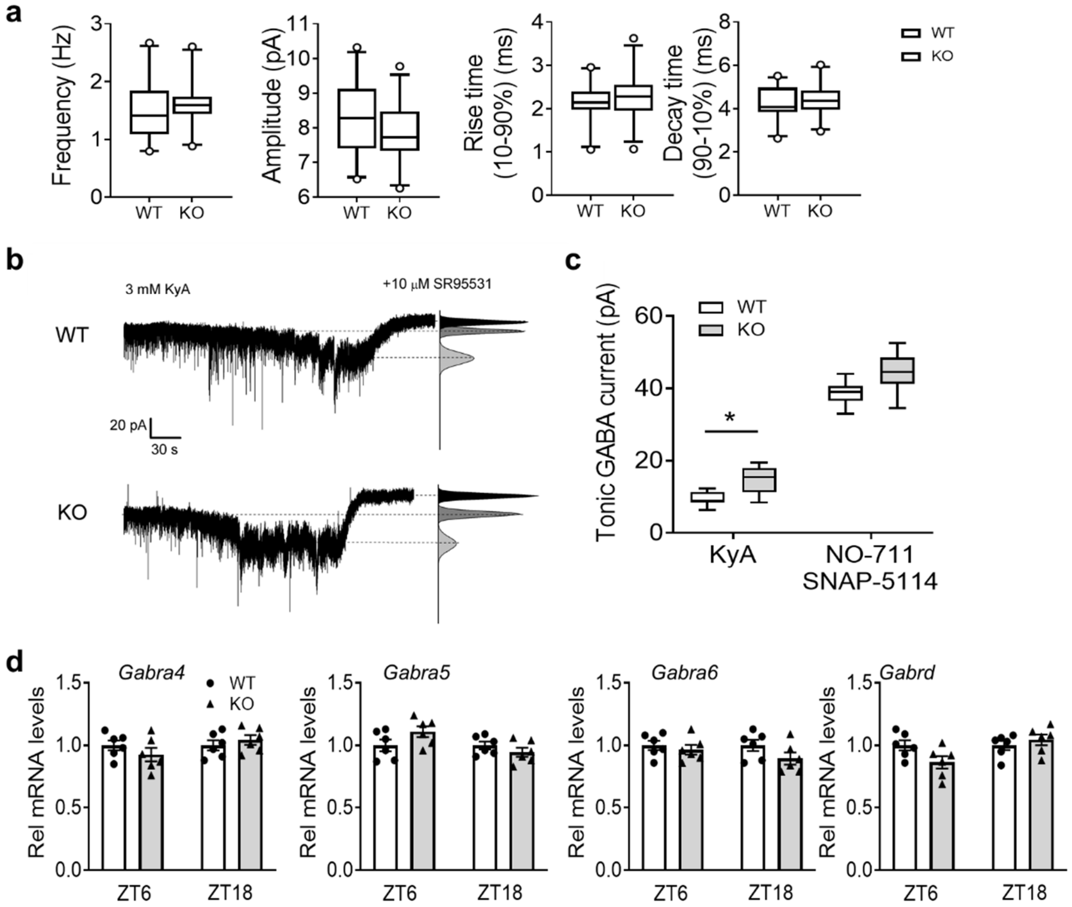

Supplementary Figure 13. Effects of Rev-erbα on miniature excitatory postsynaptic current (mEPSC) and tonic GABA current. (**a**) mEPSC frequency, amplitude, rise time and decay time derived from the dentate gyrus granule cells (DGGCs) of *Rev-erbα*$^{-/-}$ (KO) (24 cells, 5 mice) and wild type (WT) (22 cells, 5 mice) mice. (**b**) Representative traces of tonic GABA currents derived from the DGGCs of *Rev-erbα*$^{-/-}$ and WT mice. (**c**) Tonic GABA currents in DGGCs of *Rev-erbα*$^{-/-}$ (14 cells, 4 mice) and WT mice (12 cells, 4 mice) in the presence or absence of NO-711 and SNAP-5114 (two specific Slc6a1 and Slc6a11 inhibitors). p = 0.0002 (two-sided Mann-Whitney test). (**d**) mRNA expressions of GABA receptors in hippocampus of *Rev-erbα*$^{-/-}$ (KO) and wild-type (WT) mice at ZT6 and ZT18. Data are mean ± SEM (n = 6 biologically independent samples). In panels **a** and **c**, data are shown as box-and-whisker with median (middle line), 25th-75th percentiles (box), and 5th and 95th percentile (whiskers) as well as outliers (single points). *represents a p value of < 0.05. ZT, zeitgeber time.

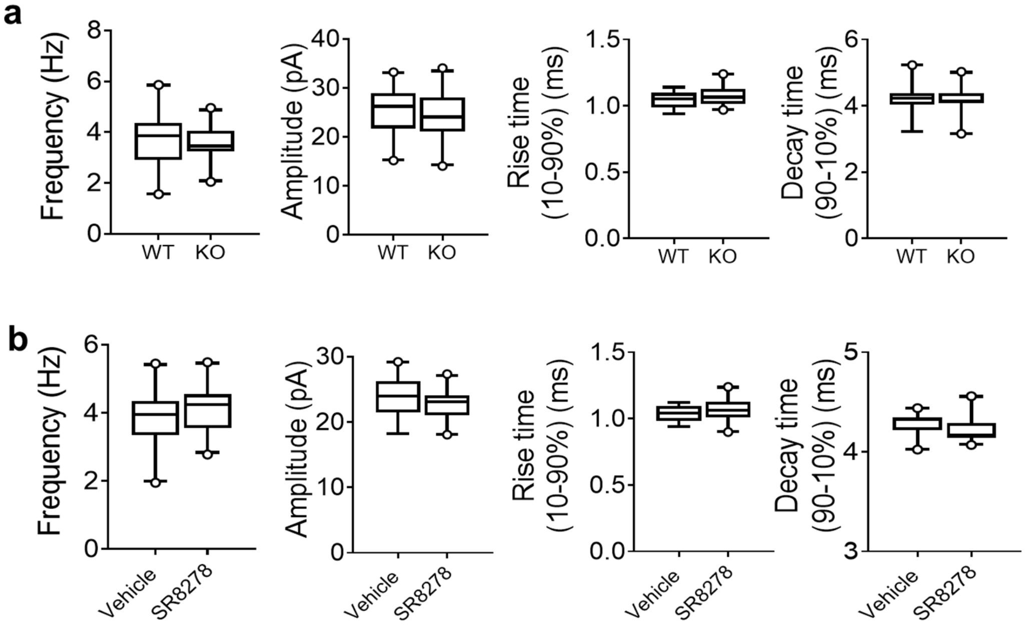

Supplementary Figure 14. **Effects of Rev-erbα ablation or antagonism on sIPSCs in the presence of Sla6a1 and Slc6a11 inhibitors.** (**a**) sIPSC frequency, amplitude, rise time and decay time derived from dentate gyrus granule cells of *Rev-erbα*$^{-/-}$ (KO) (23 cells, 5 mice) and wild-type (WT) mice (20 cells, 5 mice). (**b)** sIPSC frequency, amplitude, rise time and decay time derived from dentate gyrus granule cells of SR8278 treated mice (23 cells, 5 mice) and vehicle-treated mice (21 cells, 4 mice). All data were shown as box-and-whisker with median (middle line), 25th-75th percentiles (box), and 5th and 95th percentile (whiskers) as well as outliers (single points).

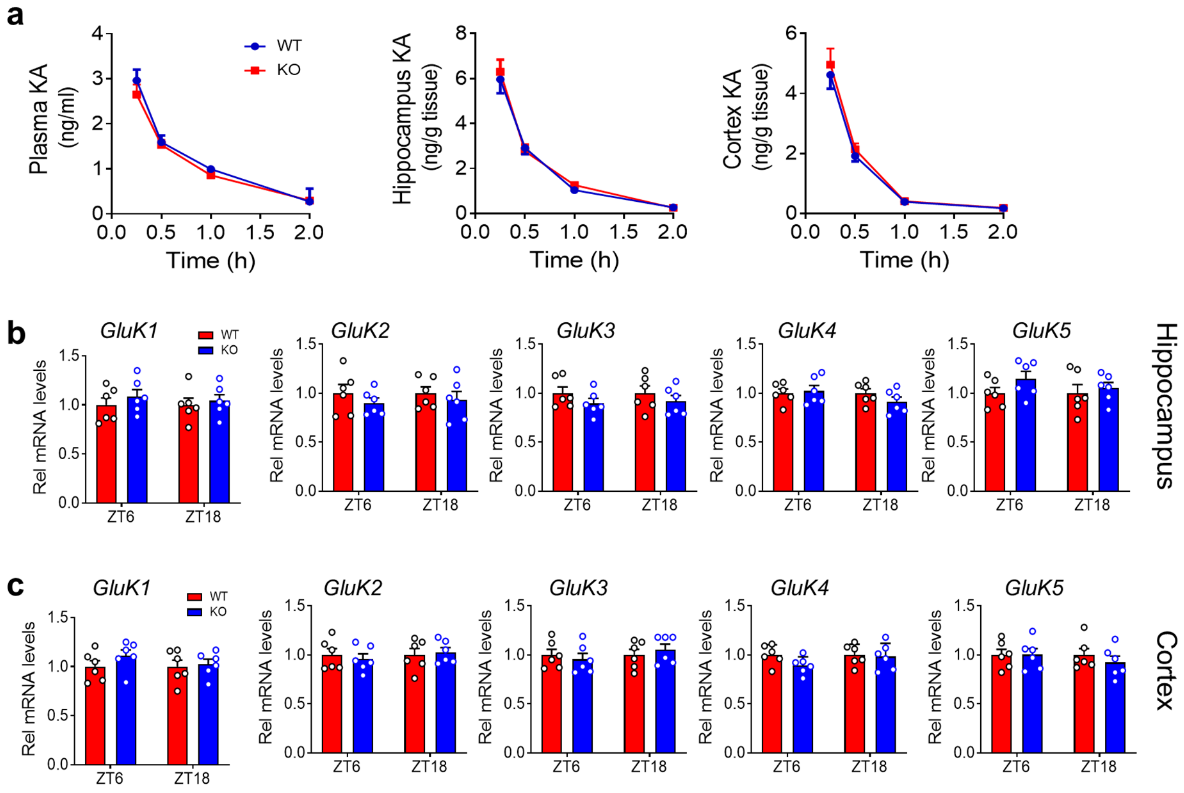

Supplementary Figure 15. Effects of Rev-erbα on KA pharmacokinetics, brain distribution, and KA receptor expression. (**a**) Plasma, hippocampus and cortex concentrations of KA in *Rev-erbα*$^{-/-}$ (KO) and wild-type (WT) mice after KA treatment (20 mg/kg, i.p., *n* = 3 mice per time point). Data are presented as mean ± SEM. (**b)** mRNA expressions of KA receptors (GluK1, GluK2, GluK3, GluK4 and GluK5) in the hippocampus of KO and WT mice at ZT6 and ZT18. (**c)** mRNA expressions of KA receptors (GluK1, GluK2, GluK3, GluK4 and GluK5) in the cortex of KO and WT mice at ZT6 and ZT18. In panels b and c, data are mean ± SEM (*n* = 6 biologically independent samples). KA, kainic acid. Rel, relative; ZT, zeitgeber time.

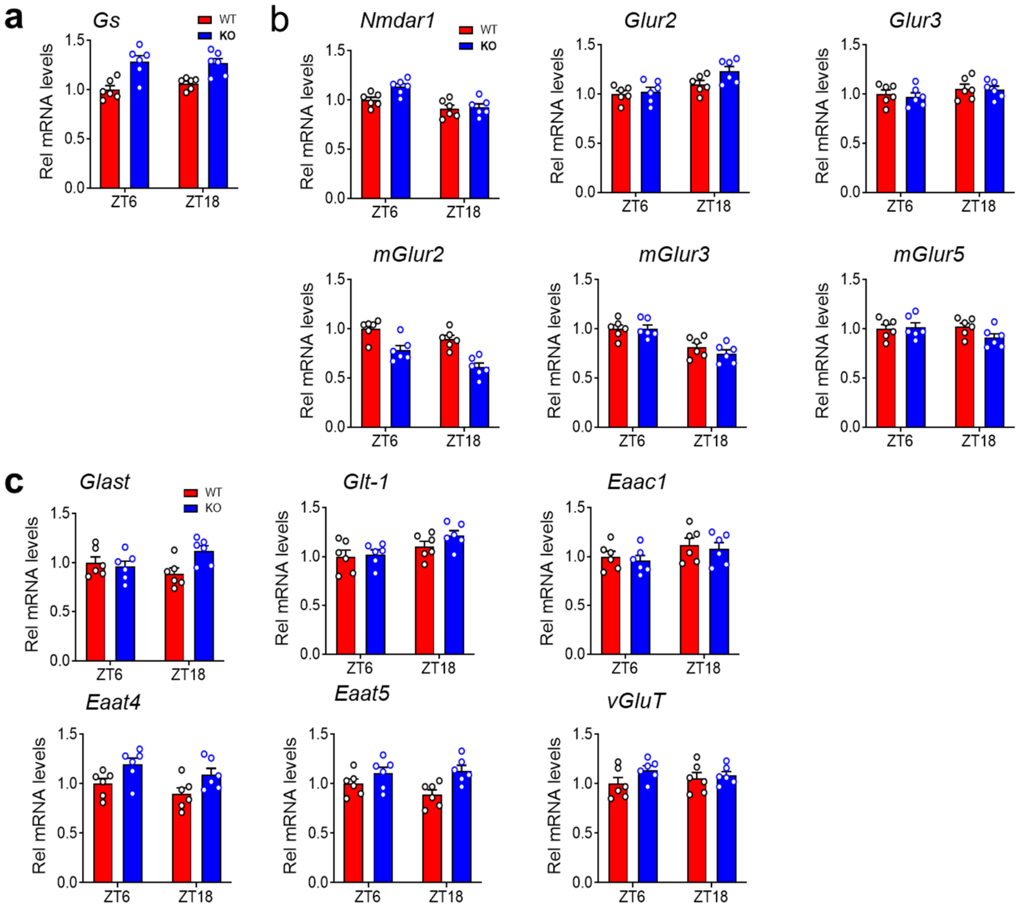

Supplementary Figure 16. **Effects of Rev-erbα ablation on expressions of glutamate-related genes in mouse hippocampus.** (**a**) mRNA expressions of *Gs* in hippocampus of *Rev-erbα*$^{-/-}$ (KO) and wild-type (WT) mice at ZT6 and ZT18. (**b**) mRNA expressions of *Nmdar1*, *Glur2*, *Glur3, mGlur2, mGlau3* and *mGlur5* in hippocampus of *Rev-erbα*$^{-/-}$ and WT mice at ZT6 and ZT18. (**c**) mRNA expressions of *Glast*, *Glt-1*, *Eaac1, Eaa4*, *Eaa5* and vGluT in hippocampus of *Rev-erbα*$^{-/-}$ and WT mice at ZT6 and ZT18. All data are shown as mean ± SEM (*n* = 6 biologically independent samples). Rel, relative; ZT, zeitgeber time.

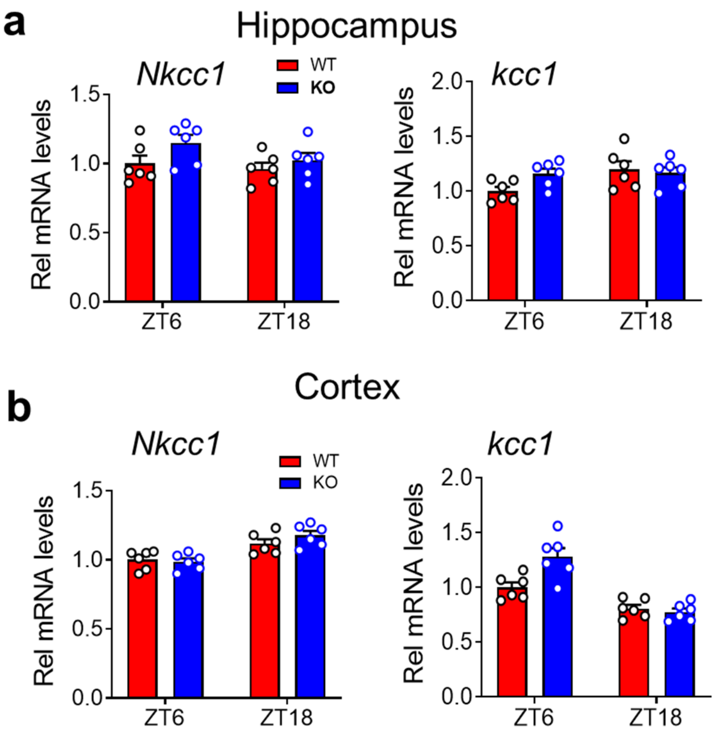

Supplementary Figure 17. **Effects of Rev-erbα on mRNA expressions of the ion channels *Nkcc1* and *Kcc1* in hippocampus (a) and cortex (b) of *Rev-erbα*$^{-/-}$ (KO) and wild-type (WT) mice at ZT6 and ZT18.** All data are shown as mean ± SEM (*n* = 6 biologically independent samples). Rel, relative; ZT, zeitgeber time.

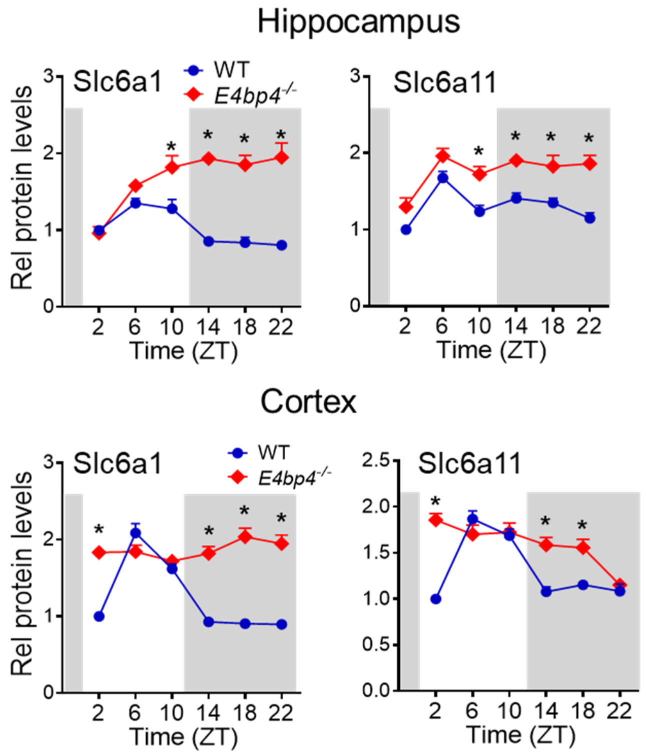

Supplementary Figure 18. Quantification data generated from Western blots in Figure 7b. *p* values (hippocampus, Slc6a1, from left to right): 0.7108, 0.0782, 0.0496, 0.0002, 0.0019, 0.0041; *p* values (hippocampus, Slc6a11, from left to right): 0.0834, 0.0914, 0.0201, 0.0098, 0.1112, 0.0252; *p* values (cortex, Slc6a1, from left to right): 0.0005, 0.1792, 0.3597, 0.0010, 0.0006, 0.0010; *p* values (cortex, Slc6a11, from left to right): 0.0005, 0.2705, 0.7884, 0.0064, 0.0156, 0.4693. *p* values were obtained from two-way ANOVA and Bonferroni post hoc test. Data are mean ± SEM, *n* = 6 mice per group. *represents a *p* value of < 0.05. Rel, relative; ZT, zeitgeber time.

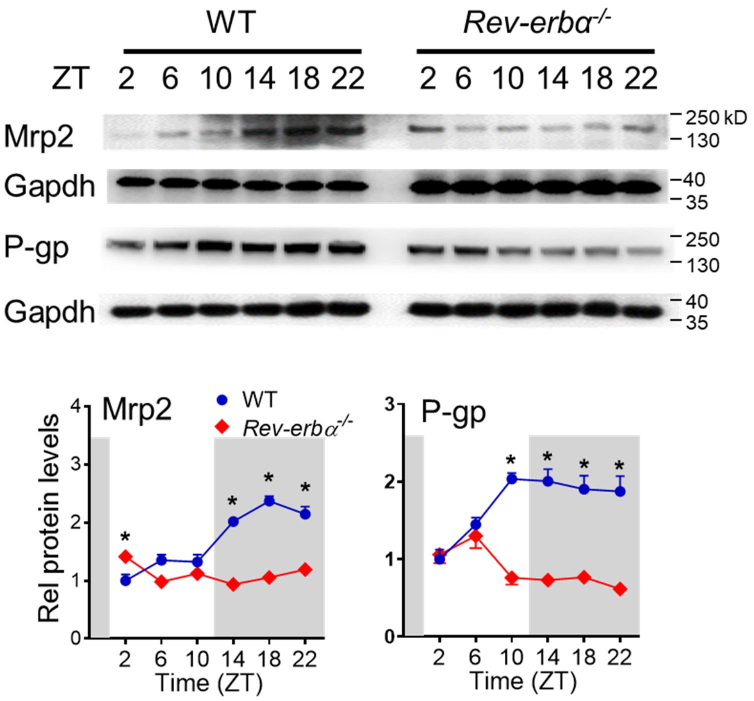

Supplementary Figure 19. Effects of Rev-erbα ablation on expressions of the efflux transporters P-gp and Mrp2 in hippocampus of mice. Western blot strips (a target protein and a loading control) were cut from one gel. *p* values (Mrp2, from left to right): 0.0397, 0.0611, 0.2901, 0.0002, 0.0003 and 0.0046. *p* values (P-gp, from left to right): 0.7350, 0.4589, 0.0004, 0.0012, 0.0031 and 0.0035. *p* values were obtained from two-way ANOVA and Bonferroni post hoc test. Data are shown as mean ± SEM, *n* = 6 mice per group. *represents a *p* value of < 0.05. Rel, relative; ZT, zeitgeber time.

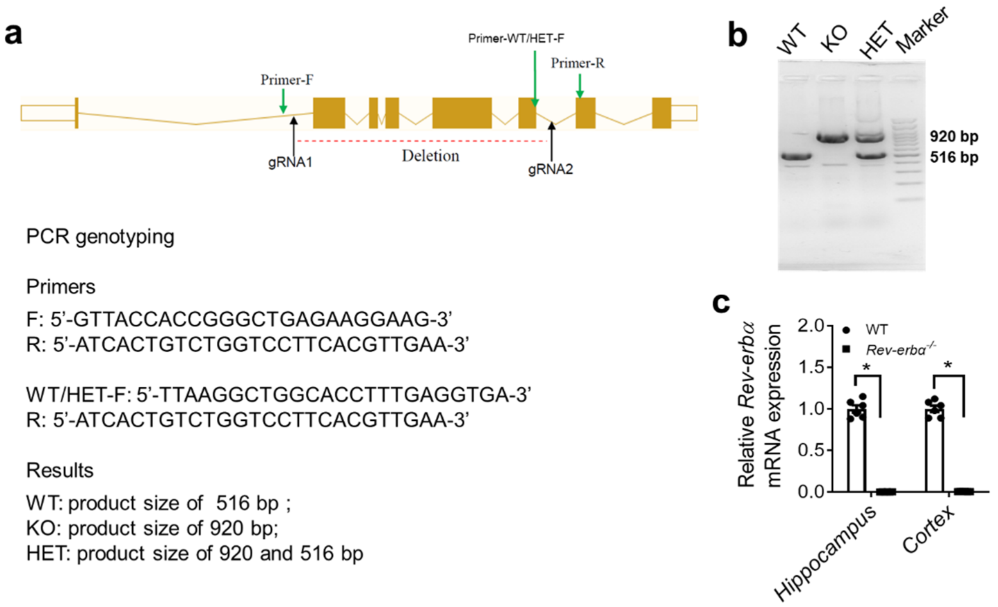

Supplementary Figure 20. Generation and characterization of *Rev-erbα*$^{-/-}$ mice. (**a**) Schematic diagram of gene sequence, showing the deleted regions in *Rev-erbα*$^{-/-}$ mice. (**b**) Genotyping of *Rev-erbα*$^{-/-}$ mice. PCR was performed using genomic DNA extracted from mouse tails. (**c**) Expression levels of Rev-erbα in the hippocampus and cortex of WT and *Rev-erbα*$^{-/-}$ mice. Data are shown as mean ± SEM (n = 6 biologically independent samples). Two-sided t test p values: < 0.0001 and < 0.0001. *represents a p value of < 0.05.

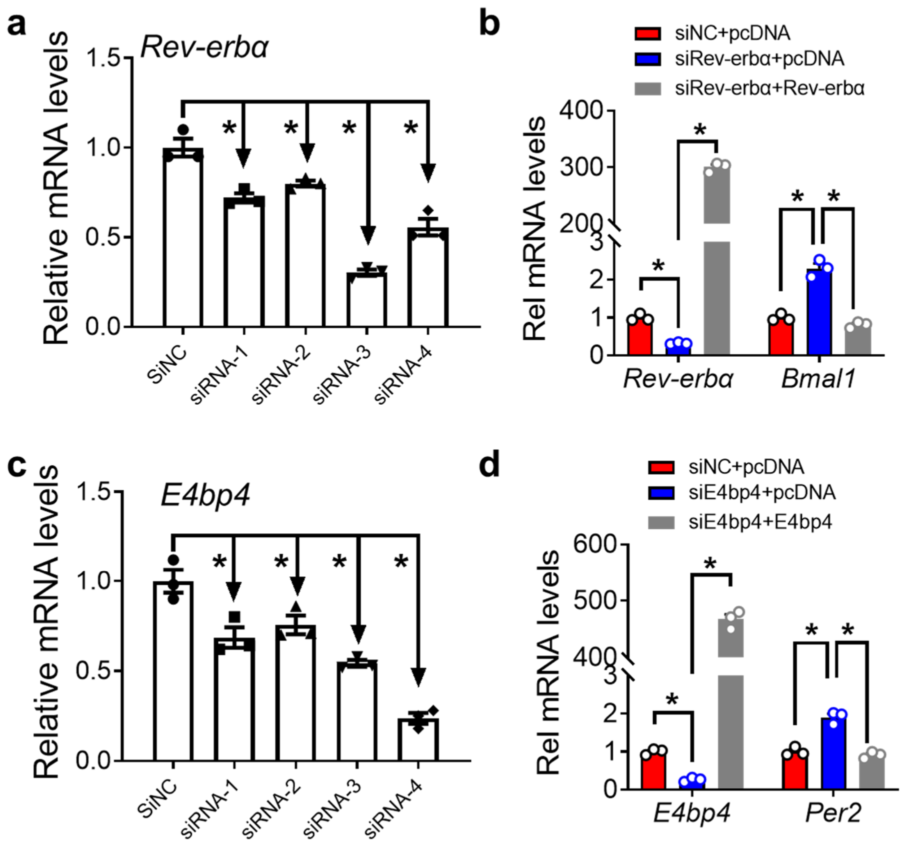

Supplementary Figure 21. **Relative efficiency of siRNAs in gene silencing and verification of siRNA specificity by rescue experiment.** (**a**) Relative efficiency of four siRNAs targeting *Rev-erbα*. *p* values (from left to right): 0.0075, 0.0194, 0.0002 and 0.0029. (**b**) Rescue experiments showing that Rev-erbα knockdown-induced change in expression of *Bmal1* (a Rev-erbα target gene) can be restored by Rev-erbα overexpression. *p* values (from left to right): 0.0003, < 0.0001, 0.0008 and 0.0004. (**c**) Relative efficiently of four siRNAs targeting *E4bp4*. *p* values (from left to right): 0.0218, 0.042, 0.0024 and 0.0004. (**d**) Rescue experiments showing that E4bp4 knockdown-induced change in expression of *Per2* (an E4bp4 target gene) can be restored by E4bp4 overexpression. *p* values (from left to right): 0.0002, < 0.0001, 0.0014 and 0.0007. All data are shown as mean ± SEM (*n* = 3 biologically independent samples). *p* values were obtained from one-way ANOVA and Bonferroni post hoc test. *represents a *p* value of < 0.05. Rel, relative.
