# Supplementary material for: Dysregulation of REV-ERBα impairs GABAergic function and promotes epileptic seizures in preclinical models
Source: Nat Commun. 2021 Feb 22;12:1216. doi: 10.1038/s41467-021-21477-w (PMC7900242; doi:10.1038/s41467-021-21477-w)
Supplement: Supplementary file 3 — Reporting Summary [file 41467_2021_21477_MOESM3_ESM.pdf]

nature research

Corresponding author(s): Baojian Wu and Jiandong Yu

Last updated by author(s): Jan 18, 2021

Reporting Summary

Nature Research wishes to improve the reproducibility of the work that we publish. This form provides structure for consistency and transparency in reporting. For further information on Nature Research policies, see our Editorial Policies and the Editorial Policy Checklist.

Statistics

For all statistical analyses, confirm that the following items are present in the figure legend, table legend, main text, or Methods section.

n/a	Confirmed	
☐	☒	The exact sample size (n) for each experimental group/condition, given as a discrete number and unit of measurement
☐	☒	A statement on whether measurements were taken from distinct samples or whether the same sample was measured repeatedly
☐	☒	The statistical test(s) used AND whether they are one- or two-sided<br>*Only common tests should be described solely by name; describe more complex techniques in the Methods section.*
☐	☒	A description of all covariates tested
☐	☒	A description of any assumptions or corrections, such as tests of normality and adjustment for multiple comparisons
☐	☒	A full description of the statistical parameters including central tendency (e.g. means) or other basic estimates (e.g. regression coefficient) AND variation (e.g. standard deviation) or associated estimates of uncertainty (e.g. confidence intervals)
☐	☒	For null hypothesis testing, the test statistic (e.g. F, t, r) with confidence intervals, effect sizes, degrees of freedom and P value noted<br>*Give P values as exact values whenever suitable.*
☒	☐	For Bayesian analysis, information on the choice of priors and Markov chain Monte Carlo settings
☒	☐	For hierarchical and complex designs, identification of the appropriate level for tests and full reporting of outcomes
☒	☐	Estimates of effect sizes (e.g. Cohen's d, Pearson's r), indicating how they were calculated

Our web collection on statistics for biologists contains articles on many of the points above.

Software and code

Policy information about availability of computer code

Data collection	PAL 8200 software v1.10.4
Data analysis	HISAT2 v2.0.4; Cuffdiff v2.0.1; GraphPad Prism (7.0); Origin software 8.0; Clampfit software 9.0; Quantity One software 4.62; ImageJ software 1.8.

For manuscripts utilizing custom algorithms or software that are central to the research but not yet described in published literature, software must be made available to editors and reviewers. We strongly encourage code deposition in a community repository (e.g. GitHub). See the Nature Research guidelines for submitting code & software for further information.

Data

Policy information about availability of data

All manuscripts must include a data availability statement. This statement should provide the following information, where applicable:

- Accession codes, unique identifiers, or web links for publicly available datasets
- A list of figures that have associated raw data
- A description of any restrictions on data availability

RNA-seq data have been deposited to Sequence Read Archive (SRA) under the accession number PRJNA637449 (https://www.ncbi.nlm.nih.gov/bioproject/PRJNA637449). Other data supporting the findings of this study are available within the paper and its supplementary information files. Source data are provided as a Source Data file.

Field-specific reporting

Please select the one below that is the best fit for your research. If you are not sure, read the appropriate sections before making your selection.

- [x] Life sciences
- [ ] Behavioural & social sciences
- [ ] Ecological, evolutionary & environmental sciences

For a reference copy of the document with all sections, see nature.com/documents/nr-reporting-summary-flat.pdf

Life sciences study design

All studies must disclose on these points even when the disclosure is negative.

Sample size	Sample sizes were estimated based on previously published studies [Li et al. Neuron 2017;96(2):387-401; Wang et al. Neuron 2017;95 (5):1221; Jimenez-Mateos et al. Nat Med 2012;18(7):1087-94; Zhang T et al. Hepatology 2019 ;70(5):1770-1784; Wang S et al. Nat Commun 2018;9(1):4246]. No statistical test was performed to predetermine sample size. Sample sizes may vary depending on sample availability.
Data exclusions	No data were excluded from the analyses.
Replication	All experiments were repeated with reproducibility. The replication number for each experiment is indicated in the legend of the corresponding figure. All independent scores indicated in the figures are biologically independent. At least three independent experiments at different dates were performed to verify the findings in vivo or in vitro.
Randomization	For in vivo studies, the animals were randomly allocated to experimental groups. For in vitro studies, the cells from each cell line required for all tested conditions were pooled, equal number of cell were then seeded and treated randomly.
Blinding	For mouse experiments (including survival, behavioral and RNA-seq analysis) , the identity (animal genotype or treatment condition) of each mouse was blinded when measurements were collected (the investigators were blinded to group allocation during data collection). The quantification of immunoblotting and immunofluorescence data was performed by three independent lab personnel that were blinded for analysis (the investigators were blinded to group allocation during data collection). For other in vitro experiments, the investigators were not blinded for group allocation as the same investigators both planned and performed the experiments.

Reporting for specific materials, systems and methods

We require information from authors about some types of materials, experimental systems and methods used in many studies. Here, indicate whether each material, system or method listed is relevant to your study. If you are not sure if a list item applies to your research, read the appropriate section before selecting a response.

Materials & experimental systems

n/a	Involved in the study	
[ ]	[x]	Antibodies
[ ]	[x]	Eukaryotic cell lines
[x]	[ ]	Palaeontology and archaeology
[ ]	[x]	Animals and other organisms
[ ]	[x]	Human research participants
[x]	[ ]	Clinical data
[x]	[ ]	Dual use research of concern

Methods

n/a	Involved in the study	
[x]	[ ]	ChIP-seq
[x]	[ ]	Flow cytometry
[ ]	[x]	MRI-based neuroimaging

Antibodies

Antibodies used	REV-ERBα (14506-1-AP, Proteintech, 1:1000 for WB), SLC6A1 (A15099, Abclonal, 1:500 for WB), SLC6A11 (A11702, Abclonal, 1:500 for WB), BMAL1 (ab3350, Abcam, 1:200 for WB), DBP (ab22824, Abcam, 1:1000 for WB), CLOCK (ab3517, Abcam, 1:200 for WB), E4BP4 (M225-3, MBL, 1:500 for WB), REV-ERBα (ab174309, Abcam, 1:100 for IHC), GFAP (ab68428, Abcam, 1:500 for IHC), NeuN (ab104224, Abcam, 1:500 for IHC), Iab1 (ab48004, Abcam, 1:200 for IHC), rabbit IgG-HRP secondary antibody (HA1001, HuaBio, 1:5000), rabbit Alexa Fluor 488 secondary antibody (A-11034, Thermo Fischer, 1:400), mouse Alexa 555 secondary antibody (A-31570, Thermo Fischer, 1:200), Goat Alexa 555 secondary antibody (A-21432, Thermo Fischer, 1:400).
Validation	(1) REV-ERBα: human, mouse; WB, IP, IF, chIP, ELISA.(https://www.ptgcn.com/products/nr1d1-antibody-14506-1-ap.htm)<br>(2) SLC6A1: human, mouse, rat; WB, IF. (https://abclonal.com/catalog-antibodies/SLC6A1PolyclonalAntibody/A15099)<br>(3) SLC6A11: human, mouse, rat; WB. (https://abclonal.com/catalog-antibodies/SLC6A11PolyclonalAntibody/A11702)<br>(4) BMAL1: human, mouse, hamster; WB, IF/ICC. (https://www.abcam.cn/bmal1-antibody-ab3350.html)<br>(5) DBP: human, mouse; WB, IF. (Meier et al. PloS one. 2015;10(9): e0137229, Ma et al. Diabetologia. 2016;59(2): 354-362, Yu et al. Theranostics. 2019;9(10): 2754.) (https://www.abcam.cn/d-box-binding-protein-antibody-bsa-free-ab22824.html)<br>(6) CLOCK: human, mouse, hamster; WB, ICC, IHC-P. (https://www.abcam.cn/kat13d--clock-antibody-ab3517.html)<br>(7) E4BP4: human, mouse; WB, IP, IH, FCM. (https://www.mblbio.com/bio/g/dtl/A/?pcd=M225-3)<br>(8) REV-ERBα: mouse, rat, human; ICC/IF, Flow Cyt, IHC-P, WB. (https://www.abcam.cn/nr1d1-antibody-epr10376-ab174309.html)

(9) GFAP: mouse, rat, human; WB, IP, IHC-P, mIHC, ICC, IHC-F. (https://www.abcam.com/gfap-antibody-epr1034y-ab68428.html)
(10) NeuN: mouse, rat, human; ICC, IHC-P, WB. (https://www.abcam.com/neun-antibody-1b7-neuronal-marker-ab104224.html)
(11) Iba1: mouse, human; WB, Flow Cyt, ICC/IF, IHC-P. (https://www.abcam.com/iba1-antibody-ab48004.html)

Eukaryotic cell lines

Policy information about cell lines

Cell line source(s)	Neuro-2a cells were obtained from ATCC.
Authentication	Cells from ATCC were recognized, no further authentication.
Mycoplasma contamination	No mycoplasma testing was performed.
Commonly misidentified lines (See ICLAC register)	No commonly misidentified lines were used.

Animals and other organisms

Policy information about studies involving animals; ARRIVE guidelines recommended for reporting animal research

Laboratory animals	Wild-type C57BL/6 (male, age of 8-12 weeks), and Rev-erbα-/-, Rev-erbβ-/- and E4bp4-/- mice (male, age of 8-12 weeks, on a C57BL/6 background) were used in this study. Mice were housed under a 12 h light/dark cycle at controlled room temperature of 22-25°C and a relative humidity of 40-60%.
Wild animals	No wild animals were used in this study.
Field-collected samples	No field-collected samples were used in this study.
Ethics oversight	The animal experiments were approved by the Ethics Committee of Jinan University and the experimental procedures strictly followed the guidelines for Institutional Animal Care and Use.

Note that full information on the approval of the study protocol must also be provided in the manuscript.

Human research participants

Policy information about studies involving human research participants

Population characteristics	The information of participants is provided in Supplementary Table 2.
Recruitment	Human samples were obtained from the First Affiliated Hospital of Jinan University. The TLE and glioma (as a control) patients were recruited to assess deregulation of REV-ERBα in TLE. For TLE group of patients, epileptogenic foci were identified by brain PET-CT, MRI scan and EEG. For control group, glioma patients without seizure occurrence were recruited. The surgical specimens were collected from therapeutic resections for treatment of epilepsy or glioma.<br>We do not expect any bias during recruitment as the biospecimens were analyzed by independent researchers.
Ethics oversight	The study protocol was approved by the institutional review board of the First Affiliated Hospital of Jinan University.

Note that full information on the approval of the study protocol must also be provided in the manuscript.

Magnetic resonance imaging

Experimental design

Design type	Clinical MRI was performed to confirm epileptogenic foci in patients with TLE
Design specifications	Brain MRI obtained from axial, coronal and sagittal planes.
Behavioral performance measures	No behavioral performance measures were performed.

Acquisition

Imaging type(s)	Structural MRI.
Field strength	3.0T
Sequence & imaging parameters	Maltiplanner multisquential (T1W; T2W TSE). Anatomical imaging: Fov read, 210 mm; Fov phase, 100 percent; Repetition time (TR), 3000 ms; Echo time (TE), 90 ms; Flip angle, 150 degree; Slice thickness (3 mm).
Area of acquisition	Whole brain

Diffusion MRI ☐ Used ☒ Not used

Preprocessing

Preprocessing software	Syngo MR B17
Normalization	No normalization was used.
Normalization template	No spatial normalization/transformation was used
Noise and artifact removal	No artifact or noise removal was used.
Volume censoring	No volume censoring was used.

Statistical modeling & inference

Model type and settings	No statistical modelling or inference was used
Effect(s) tested	Not relevant. We did not perform functional magnetic resonance imaging analyses, thus no effects were tested.

Specify type of analysis: ☒ Whole brain ☐ ROI-based ☐ Both

Statistic type for inference (See Eklund et al. 2016)	NA
Correction	NA

Models & analysis

n/a	Involved in the study
☒	☐ Functional and/or effective connectivity
☒	☐ Graph analysis
☒	☐ Multivariate modeling or predictive analysis
